# SOX2 haploinsufficiency is associated with slow progressing hypothalamo-pituitary tumours

**DOI:** 10.1002/humu.21606

**Published:** 2011-09-12

**Authors:** Kyriaki S Alatzoglou, Cynthia L Andoniadou, Daniel Kelberman, Charles R Buchanan, John Crolla, Maria Cristina Arriazu, Martin Roubicek, Daniel Moncet, Juan P Martinez-Barbera, Mehul T Dattani

**Affiliations:** 1 Developmental Endocrinology Research Group, Clinical and Molecular Genetics Unit, Institute of Child Health, University College LondonLondon, United Kingdom; 2 Neural Development Unit, Institute of Child Health, University College LondonLondon, United Kingdom; 3 Ulverscroft Vision Research Group, Developmental Biology Unit, Institute of Child Health, University College LondonLondon, United Kingdom; 4 Department of Child Health, Kings College Hospital NHS Foundation TrustLondon, United Kingdom; 5 Wessex Regional Genetics Laboratory, Salisbury District HospitalSalisbury, United Kingdom; 6 Department of Pediatric Endocrinology, Hospital Privado de ComunidadBuenos Aires, Argentina

**Keywords:** SOX2, pituitary, tumors, ß-catenin

## Abstract

SOX2 is an early developmental transcription factor and marker of stem cells that has recently been implicated in the development of the pituitary gland. Heterozygous *SOX2* mutations have been described in patients with hypopituitarism and severe ocular abnormalities. In the majority of published cases, the pituitary gland is either small or normal in size. Here, we report two unrelated patients with *SOX2* haploinsufficiency (a heterozygous gene deletion and a novel c.143TC>AA/p.F48X mutation) who developed nonprogressive pituitary tumors of early onset, suggesting a congenital etiology. The truncating mutation resulted in significant loss of function and impaired nuclear localization of the mutant protein, in addition to a failure to repress β-catenin transcriptional activity in vitro. This is the first indication that *SOX2* haploinsufficiency is implicated in the generation of pituitary tumors with distinct clinical characteristics, possibly mediated via its effects on the Wnt signaling pathway. 32:1376–1380, 2011. ©2011 Wiley Periodicals, Inc.

SOX2 (MIM# 184429), a member of the SOXB1 family of transcription factors, is a widely expressed marker of progenitor and stem cells. The single exon gene encodes a 317 amino acid protein that contains an N-terminal domain, a DNA-binding high-mobility group (HMG) domain and a C-terminal transcriptional activation domain [Collignon et al., 1996]. *SOX2* haploinsufficiency in both mouse and human has been associated with variable hypopituitarism associated with anterior pituitary hypoplasia, suggesting that it has a critical role in the development of the anterior pituitary [Kelberman et al., 2006]. Heterozygous de novo mutations in humans are associated with severe ocular phenotypes (bilateral anophthalmia or severe microphthalmia) and hypogonadotropic hypogonadism (HH) with or without associated abnormalities such as esophageal atresia, male genital anomalies, developmental delay, sensorineural deafness, hippocampal malformation, hypoplasia of the corpus callosum, and hypothalamic hamartoma [Kelberman et al., 2006, 2008]. In humans, *SOX2* expression is detected within Rathke's pouch and maintained throughout the development of the anterior pituitary, as well as in the presumptive hypothalamus and neural ectoderm [Kelberman et al., 2008]. In neural progenitors, SOX2 downregulation is associated with progression from a proliferating undifferentiated state to a committed phenotype [Graham et al., 2003]. In the murine adult pituitary, SOX2 expression is maintained in a small population of cells lining the pituitary cleft, which show many of the properties of progenitor cells and have the ability to differentiate into all hormone-producing cell types [Fauquier et al., 2008].

The specificity of the action of SOX proteins depends largely on their interaction with partner proteins. Recent data have suggested an interaction with components of another early developmental pathway, the Wnt/β-catenin signaling pathway. Members of the Wnt/β-catenin pathway are expressed in the developing pituitary and are implicated in the maintenance of normal morphology of the gland and the determination of hormone-secreting cell types [Olson et al., 2006; Potok et al., 2008]. *Xenopus* XSox3 and XSox17 as well as murine SOX2 interact with β-catenin and repress its activity in vitro [Mansukhani et al., 2005; Zorn et al., 1999]. We have shown that human SOX2 is also capable of inhibiting β-catenin-mediated transcriptional activation [Kelberman et al., 2008]. Recent studies have suggested that aberrant activation of the Wnt pathway, resulting from sustained β-catenin activation or downregulation of Wnt-inhibitors is associated with the development of pituitary tumors [Buslei et al., 2005; Elston et al., 2008; Gaston-Massuet et al., 2011]. Here we report, for the first time to our knowledge, the identification of heterozygous *SOX2* mutations in two unrelated patients in association with pituitary tumors of likely congenital origin and we provide in vitro evidence that disruption of the SOX2/β-catenin interaction may be the molecular mechanism underlying some human pituitary tumors.

Case 1 is a female patient with bilateral anophthalmia who presented for the first time at the age of 18 years for assessment of pubertal delay. She was the second child of nonconsanguineous parents born at term with a birth weight of −1.0 standard deviation scor (SDS), and had severely impaired language development and delayed motor milestones. At presentation, she was prepubertal (Tanner staging 1) with a height of 144.8 cm (−3.12 SDS). Basal endocrine investigations demonstrated undetectable estradiol with low basal gonadotropins and a flat luteinising hormone (LH) and follicle stimulating hormone (FSH) response to GnRH stimulation confirming a diagnosis of HH (Table 1). Magnetic resonance imaging (MRI) revealed a sellar tumor with a cystic component, extending into the suprasellar area (Fig. 1A), without evidence of compression syndrome. Hormone replacement treatment was declined and at the age of 24 years, she went on to develop spontaneous but incomplete puberty (breast Tanner stage 2). Repeat MRI at that stage as well as sequential MR imaging over a period of 10 years did not show any significant change in the size or morphology of the tumor (Fig. 1B). There was no evidence of development of additional pituitary hormone deficiencies (Table 1) and the patient has been managed conservatively.

**Figure 1:**
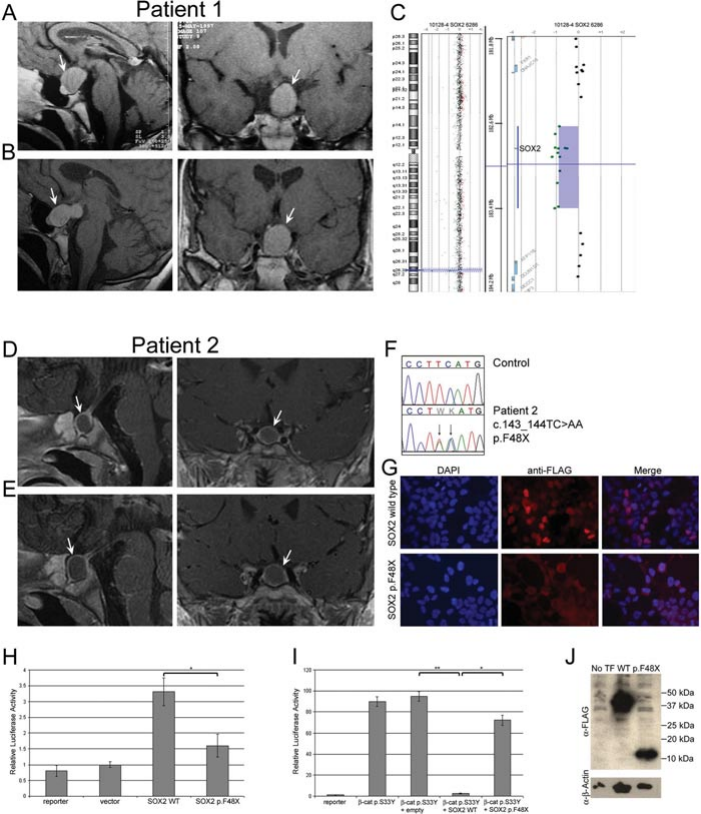
Identification and characterization of novel *SOX2* heterozygous mutations in association with slow-growing sellar tumors. (A and B) Midline sagittal (left) and coronal (right) MRI scans of patient 1 at 18 years (A) and 28 years of age (B) revealing a large mass in the location of the pituitary gland (arrows). (C) Array-CGH profile from patient 1 showing the ratio of probes, each represented by a single dot plotted as a function of chromosome position; loss of copy number of a probe shifts the ratio to the left showing deletion of *SOX2*. (D and E) Midline sagittal (left) and coronal (right) MRI scans of patient 2 at 17 months (D) and at 32 months of age (E) showing a large mass in the location of the pituitary gland (arrows). (F) Sequence electropherogram showing a two-base substitution c.143_144TT>AA (p.F48X) in patient 2. Nucleotide numbering reflects cDNA with +1 corresponding to the A of the ATG translation initiation codon in the reference sequence. (G) Overexpression of FLAG-tagged SOX2 p.F48X in HEK293T cells results in impaired localization of the protein whereas FLAG-tagged wild type SOX2 localizes in the nucleus. Nuclei are counterstained with DAPI. (H) Luciferase assay using the *Hesx1* minimal promoter demonstrates impaired activation of the promoter through expression of SOX2 p.F48X (*t*-test, *P* = 0.0006). (I) Luciferase assay using the TOPFLASH reporter reveals that expression of the SOX2 p.F48X mutant protein does not result in repression of Wnt signaling, activated by expression of a stable mutant form of β-catenin (p.S33Y) (*t*-test, *P* = 0.0002), whereas expression of wild-type SOX2 represses this activation (*t*-test, *P* < 0.0001). (J) Western blot analysis showing similar expression levels of both wild-type (WT) and p.F48X proteins. Although the intensity of the signal obtained with the α-FLAG antibody is not comparable, this is due to unequal loading of protein extracts in the gel, as shown by the loading control (α-β-Actin).

**Table 1:** Endocrine investigations in patients carrying *SOX2* heterozygous mutations

	Patient 1 (Ht *SOX2* deletion)			Patient 2 (c.143TC>AA, p.F48X)	
		
	1a	1b	normal range		normal range
Total T4 (µg/dl)	–	8.98	4.5–12.5		
FT4 (ng/dl)	1.40		0.9–1.9	1.24	0.7–1.94
TSH (mU/L)	1.41	1.68	0.4–4.5	–	
IGF-1 (µg/L)	270.7	291	96–502	48	51–303
IGFBP3 (mg/L)				1.42	0.80–3.90
Random GH (ng/ml)	6.84	0.60	–	–	–
Basal cortisol (µg/dl)	13.70	22.51	5–25	18.05	5–25
Peak cortisol (µg/dl)	–	–		27.80	
Basal LH (U/L)	0.19	0.10			
Basal FSH (U/L)	0.31	0.47			
Estradiol (pg/ml)	25.60	9.16			
Basal testosterone (µg/L)	–	–	–	0.20	–
Testosterone at 3–week HCG test (µg/L)				1.73	

Case 2 is a male infant, diagnosed at birth with bilateral anophthalmia, who was referred to the pediatric endocrine department at the age of 17 months for investigation of micropenis. He was the first child of nonconsanguineous parents, born at term with a birth weight of 2.83 kg (−1.7 SDS). At the time of presentation, his weight was 8.6 kg (−2.59 SDS) with a length of 74.8 cm (−2.24 SDS). He had a stretched penile length of 2.5 cm and a hypoplastic scrotum with testes of 0.5–1 ml palpable high in the scrotal sacs. Endocrine investigations revealed a normal FT4 of 1.24 ng/dl (16 pmol/L, normal range 9–25 pmol/L), peak cortisol to synacthen of 27.8 µg/dl (769 nmol/L) with a low IGF1 48 µg/L (normal range 51–303 µg/L). A 3-week hCG test demonstrated a testosterone response that was consistent with HH (1.73 µg/L, 6.0 nmol/L). MRI at that point revealed a pituitary mass with a cystic component extending to the suprasellar area (Fig. 1D). Review of an MRI scan performed in the neonatal period, which was thought to have been normal apart from absent prechiasmatic optic nerves, revealed that the mass had been present at that stage. Subsequent imaging demonstrated a modest increase in size between the age of 17 and 32 months (Fig. 1E). Based on the auxological data and the low IGF-1, he commenced treatment with rhGH (0.025 mg/kg/day) and he is under regular clinical and neuroradiological follow-up. In both cases, there was no family history of eye abnormalities, pubertal delay, or infertility.

DNA analysis using array CGH identified a heterozygous 731-kb deletion on Chr3q26 encompassing *SOX2* in patient 1 (Fig. 1C); this deletion was not identified in the unaffected mother, but paternal DNA was unavailable. Patient 2 was heterozygous for a mutation (c.143TC>AA) resulting in the substitution of phenylalanine at position 48 by a stop codon, predicted to generate a truncated protein lacking most of the HMG domain and the C-terminal domain of SOX2 (Fig. 1F). DNA from either parent was unavailable.

To characterize the functional consequences of the p.F48X truncated protein, we first analyzed the cellular localization of this mutant protein in HEK293T cells (Fig. 1G). Anti-FLAG immunostaining of cells transfected with wild-type FLAG-SOX2 revealed normal nuclear localization of the protein. However, FLAG-p.F48X protein was detected mainly in the cytoplasm, suggesting a failure of the mutant protein to concentrate in the nucleus. This is not unexpected as this mutation introduces a stop codon prior to the HMG DNA-binding domain, which contains the nuclear localization signals.

Next, we assessed the transcriptional activity of the p.F48X mutant protein on the murine *Hesx1* promoter, which contains SOX-binding sites and has been previously shown to be regulated by SOX2 [Eroshkin et al., 2002] (Fig. 1H). Transfection of plasmids expressing wild-type SOX2 led to a 3.31-fold (±0.44 SD) increase in luciferase expression levels relative to cells transfected with empty vector. In contrast, expression of the p.F48X mutant protein resulted in only a 1.59-fold (±0.36 SD) activation of the basal reporter activity (*t*-test, *P* = 0.0006). Similar effects have been reported for other SOX2 mutant proteins previously identified in patients with hypopituitarism and eye defects [Kelberman et al., 2006, 2008].

Finally, we investigated the ability of the p.F48X mutant protein to antagonize the β-catenin-mediated activation of the TOPFLASH reporter [Korinek et al., 1997] (Fig. 1I). Transfection of the constitutively active p.S33Y mutant β-catenin resulted in an 89.7-fold (±4.6 SD) activation of the basal TOPFLASH reporter activity. This transactivation of the reporter was significantly reduced when cells were cotransfected with plasmids expressing wild-type SOX2 (2.8-fold, ±0.34 SD, *t*-test, *P* < 0.0001). In contrast, cotransfection of a construct expressing the p.F48X mutant SOX2 failed to suppress the β-catenin-mediated activation of TOPFLASH compared with the wild-type SOX2 (72.3-fold, ±4.91 SD, *t*-test, *P* = 0.0002). These differences were not due to impaired synthesis of SOX2 protein, as confirmed by western blot using whole-cell extracts following transfection with wild-type and p.F48X FLAG-SOX2 (Fig 1J). Together, these experiments demonstrate that the p.F48X mutant protein is severely impaired in its transactivation activity as well as its ability to antagonise β-catenin-mediated transcriptional activation.

The possible oncogenic potential of SOX2 has been demonstrated in breast cancer [Chen et al., 2008] and its expression is upregulated in up to 23% of squamous cell lung cancers [Bass et al., 2009] as well as in small cell lung cancer [Maddison et al., 2010], squamous head and neck carcinomas [Freier et al., 2010], meningiomas [Comtesse et al., 2005], glioblastomas [Schmitz et al., 2007], pancreatic [Sanada et al., 2006], hepatocellular and bladder carcinomas, prostate cancer, and in seminomas [Schoenhals et al., 2009]. These findings are in contrast to our report whereby *SOX2* haploinsufficiency, rather than its upregulation, resulted in the development of a pituitary mass, which would suggest that SOX2 can also act as a tumor suppressor. Indeed, the oncogenic/tumor suppressor potential of *SOX2* seems to be cell-type dependent. *SOX2* expression is downregulated in gastric carcinomas [Li et al., 2004; Otsubo et al., 2008] and in regions of intestinal metaplasia in patients with Barrett's esophagus. In addition, the esophagus of hypomorphic mutant mice (*Sox2*^*EGFP/COND*^), expressing only 17% of wild-type SOX2 levels, has an appearance resembling mucus metaplasia [Que et al., 2007]. In these cell types, as in the developing pituitary, SOX2 may play a role in the control of the cell cycle through its interaction with the canonical Wnt signaling pathway. Wnt signaling plays an essential role during pituitary development in the control of proliferation of Rathke's pouch precursors and in the differentiation of the *Pouf1*-lineage (also known as *Pit1*) [Olson et al., 2006]. Overactivation of the Wnt pathway in the mouse leads to hyperplasia of the embryonic pituitary due to a significant increase in proliferation [Gaston-Massuet et al., 2008]. Moreover, overactivating mutations in β-catenin have been detected in up to 90% of adamantinomatous craniopharyngiomas (ACP) in humans [Buslei et al., 2005; Kato et al., 2004], a benign and slow-dividing rare hypothalamo-pituitary tumor, while Wnt inhibitors are downregulated in other pituitary tumors [Elston et al., 2008]. We have recently shown that mice expressing a degradation-resistant mutant β-catenin in Rathke's pouch develop pituitary tumors that closely resemble human ACP [Gaston-Massuet et al., 2011]. Our luciferase data clearly show that the p.F48X mutant protein cannot repress β-catenin-mediated transcriptional activation, which is compatible with a model whereby the two identified patients may have developed a pituitary mass during development due to increased proliferation. The nonprogressive nature of these otherwise impressive pituitary lesions supports the suggestion that they have an embryological origin. It could be postulated that in these cases failure of SOX2 to repress β-catenin activity may not be sufficient to sustain the growth of the pituitary mass and other factors (including other members of the SOX family) may be compensating for the impaired SOX2 function.

It is intriguing that only the two patients described in this study, out of many known patients with *SOX2* haploinsufficiency, developed pituitary tumors. Suprasellar lesions have so far been reported in six patients with *SOX2* mutations, including three cases of hypothalamic hamartomas [Kelberman et al., 2006; Scheider et al., 2009] and three cases of suprasellar arachnoid cysts [Kelberman et al., 2008; Scheider et al., 2009; Wang et al., 2008]. *SOX2* mutations are associated with considerable variability in phenotypes and apart from the severe ocular defects, patients rarely exhibit the full spectrum of clinical manifestations. The sensitivity of different tissues to altered dosage of SOX2 may in part explain this observation. As illustrated in the two cases reported here, it is possible that for some congenital pituitary tumors careful follow-up, rather than surgical excision, may be the treatment of choice.
