# Supplementary material for: SOX2 haploinsufficiency is associated with slow progressing hypothalamo-pituitary tumours
Source: Hum Mutat. 2011 Sep 12;32(12):1376–80. doi: 10.1002/humu.21606 (PMC3487182; doi:10.1002/humu.21606)
Supplement: Supplementary file 1 [file humu0032-1376-sd1.pdf]

Supp. Methods

Hormone Assays

Serum hormone concentrations were measured according to local protocols and normal ranges for each laboratory were taken into account.

DNA Analysis

Written informed consent for genetic analysis was obtained and approved by the local ethics committees. Genomic DNA was isolated from white cells and the single exon of SOX2 was amplified as previously described (Kelberman et al., 2008), and sequenced using BigDye v1.1 chemistry (Applied Biosystems) using a 3730XL DNA analyser. To detect chromosomal abnormalities, samples were first screened by multiple ligation probe amplification (MLPA, Holland) and any identified deletions were confirmed by the Agilent 44K Human Genome CGH microarray.

Plasmid constructs

PCR products comprising the entire *SOX2* coding region from patient 2 were generated and cloned into pcDNA3.1 (+) and pCMV/SV-Flag, the latter containing an in frame N-terminal FLAG epitope. The *Hesx1*-luciferase reporter used in this study has previously been shown to be regulated by SOX2 and to contain SOX2 binding sites (Eroshkin et al., 2002; Kelberman et al., 2006; Kelberman et al., 2008). The TOPFLASH luciferase reporter contains several TCF/LEF binding sites upstream to the firefly luciferase gene and reports β-catenin/TCF/LEF transcriptional activity (Millipore). A clone containing the full-length human β-catenin coding sequence (*CTNNB1*) was obtained from Geneservice Ltd (Cambridge, UK) (Lennon et al., 1996). To generate a constitutive active phosphorylation mutant of β-catenin, the mutation c.96C>A (p.S33Y) was introduced using the QuickChange Site directed Mutagenesis kit (Stratagene, LaJolla, CA). The pS33Y mutation removes an essential phosphorylation site that targets β-catenin for degradation and therefore, this substitution results in the stabilisation of the protein and over-activation of the TOPFLASH luciferase reporter (Korinek et al., 1997).

Transient luciferase assays using the Hesx1-luciferase and the TOPFLASH-luciferase reporters

The transcriptional activation activities of SOX2 proteins were analysed using HEK293T cells seeded at 7.5 x 10^4 per well in a 24-well plate and co-transfected with 100 ng of *Hesx1*-luciferase reporter and increasing amounts of wild type and mutant SOX2 expression constructs (50-150ng). To investigate the effect of SOX2 proteins on the β-catenin-mediated target activation, HEK293T cells were cotransfected with 50 ng TOPFLASH reporter, 50 ng β-catenin expression construct and 100ng of wild type or mutant SOX2 construct. Co-transfection of PRL-SV40 Renilla Luciferase (Promega) was used to control for transfection efficiency. The total amount of DNA was normalised to 250ng per reaction. Cells were transfected using FuGene reagent (Roche) according to manufacturer’s instructions. Approximately 48 hours following transfection, cells were harvested and assayed for luciferase activity using the Dual-Luciferase Reporter Assay System (Promega). Luciferase activity was measured using a BMG FLUOstar

Optima multiplate reader (BMG LABTECH Gmbh, Offenburg, Germany) and experiments were repeated three times in triplicate. Data are presented as mean ± standard deviation (SD).

Cell localisation studies

A total of $1x10^5$ HEK293T cells were seeded into each well of a 12-well tissue culture plate containing gelatine-coated glass cover slips and were transfected with 200ng FLAG-tagged SOX2 expression construct (wild type or p.F48X) using the FuGene transfection reagent. 24 hours after transfection cells were washed, fixed in 4% PFA for 20 minutes on ice and immunostained with anti-FLAG M2 (1:250 dilution, Sigma) and Alexa Fluor Goat anti-Mouse 594 antibody (1:350 dilution, Molecular Probes). Nuclear counterstaining and mounting was performed with a Vectashield containing DAPI (Vector Laboratories). Cells were visualised on a Zeiss Axioskop2 microscope and images captured with a Leica DC500 camera and image software. Experiments were performed in triplicate.

Western blotting

24 hours prior to transfection, $5x10^5$ HEK293T cells were seeded into each well of a 6-well tissue culture plate. Cells were transfected with 3µg of FLAG-tagged SOX2 expression construct (wild type or p.F48X) per well. Cells were harvested 48 hours after transfection and resuspended in lysis buffer containing 0.1% Triton X. Proteins from whole cell extracts were isolated, resolved by SDS-PAGE, transferred to nitrocellulose, and blotted using an HRP-conjugated anti-FLAG antibody (Sigma, St. Louis, MO). Immunoreactive proteins were visualised using the ECL Detection Reagent System (Amersham).

References

Eroshkin F, Kazanskaya O, Martynova N, Zaraisky A. 2002. Characterization of cis-regulatory elements of the homeobox gene Xanf-1. Gene 285:279-286.

Kelberman D, de Castro SC, Huang S, Crolla JA, Palmer R, Gregory JW, Taylor D, Cavallo L, Faienza MF, Fischetto R, Achermann JC, Martinez-Barbera JP, Rizzoti K, Lovell-Badge R, Robinson IC, Gerrelli D, Dattani MT. 2008. SOX2 plays a critical role in the pituitary, forebrain, and eye during human embryonic development. J Clin Endocrinol Metab 93:1865-1873.

Kelberman D, Rizzoti K, Avilion A, Bitner-Glindzicz M, Cianfarani S, Collins J, Chong WK, Kirk JM, Achermann JC, Ross R, Carmignac D, Lovell-Badge R, Robinson IC, Dattani MT. 2006. Mutations within Sox2/SOX2 are associated with abnormalities in the hypothalamo-pituitary-gonadal axis in mice and humans. J Clin Invest 116:2442-2455.

Korinek V, Barker N, Morin PJ, van WD, de WR, Kinzler KW, Vogelstein B, Clevers H. 1997. Constitutive transcriptional activation by a beta-catenin-Tcf complex in APC-/- colon carcinoma. Science 275:1784-1787.

Lennon G, Auffray C, Polymeropoulos M, Soares MB. 1996. The I.M.A.G.E. Consortium: an integrated molecular analysis of genomes and their expression. Genomics 33:151-152.
